# The p38 pathway, a major pleiotropic cascade that transduces stress and metastatic signals in endothelial cells

**DOI:** 10.18632/oncotarget.18264

**Published:** 2017-05-29

**Authors:** Isabelle Corre, François Paris, Jacques Huot

**Affiliations:** ^1^ CRCINA, INSERM, CNRS, Université de Nantes, Nantes, France; ^2^ Le Centre de Recherche du CHU de Québec-Université Laval et le Centre de Recherche sur le Cancer de l’Université Laval, Québec, Canada

**Keywords:** p38MAPK, endothelial cells, oxidative stress, cancer, endothelial dysfunction

## Abstract

By gating the traffic of molecules and cells across the vessel wall, endothelial cells play a central role in regulating cardiovascular functions and systemic homeostasis and in modulating pathophysiological processes such as inflammation and immunity. Accordingly, the loss of endothelial cell integrity is associated with pathological disorders that include atherosclerosis and cancer. The p38 mitogen-activated protein kinase (MAPK) cascades are major signaling pathways that regulate several functions of endothelial cells in response to exogenous and endogenous stimuli including growth factors, stress and cytokines. The p38 MAPK family contains four isoforms p38α, p38β, p38γ and p38δ that are encoded by four different genes. They are all widely expressed although to different levels in almost all human tissues. p38α/MAPK14, that is ubiquitously expressed is the prototype member of the family and is referred here as p38. It regulates the production of inflammatory mediators, and controls cell proliferation, differentiation, migration and survival. Its activation in endothelial cells leads to actin remodeling, angiogenesis, DNA damage response and thereby has major impact on cardiovascular homeostasis, and on cancer progression. In this manuscript, we review the biology of p38 in regulating endothelial functions especially in response to oxidative stress and during the metastatic process.

## INTRODUCTION

**Figure 1:**
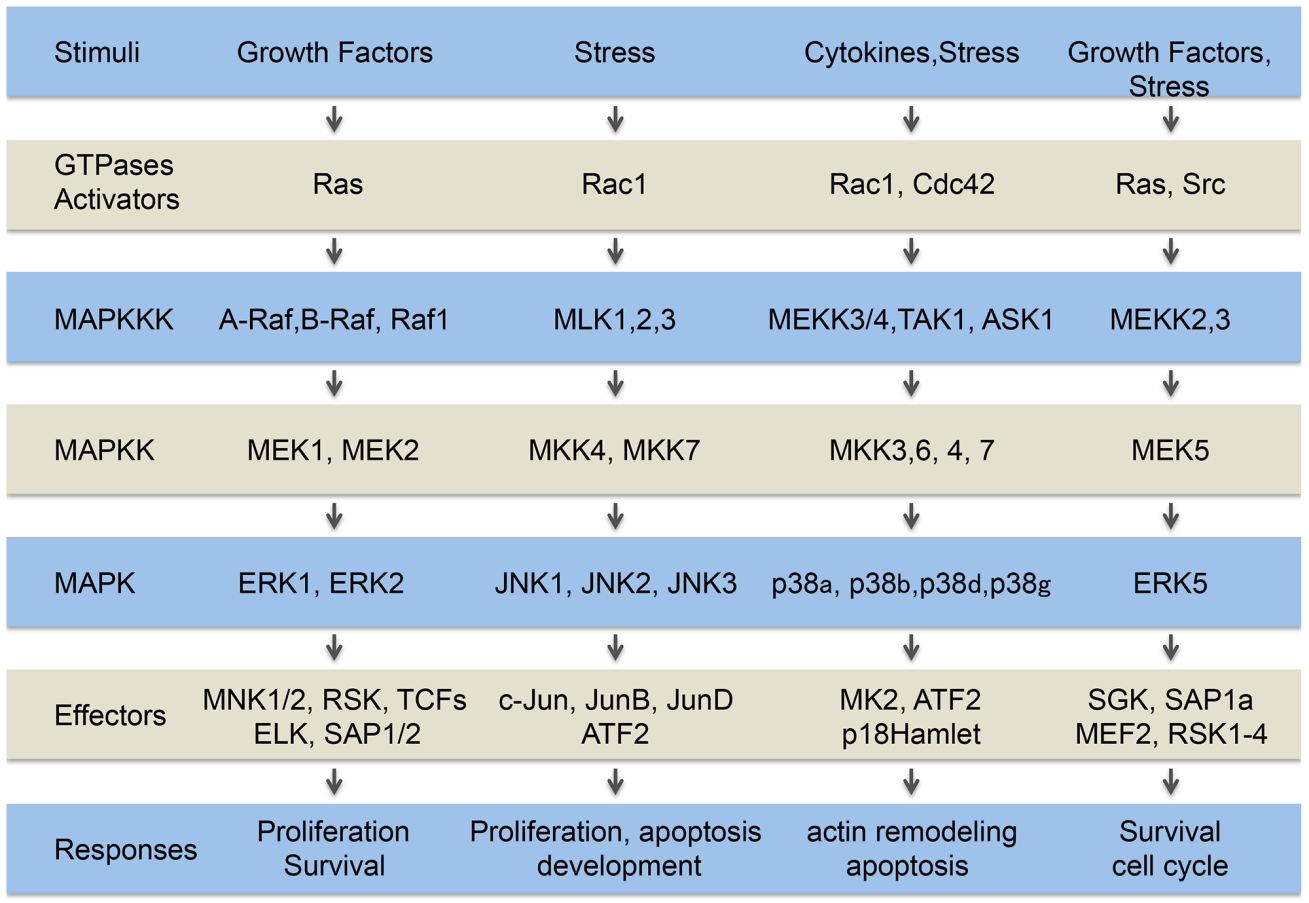
The MAP kinase pathways Extracellular signals generated by growth factors, cytokines and stresses are transduced within the cells by several pathways including the classical MAP kinase pathways, ERK1/2, ERK5 and JNK and p38 family members. The MAP kinases are constituted of a MAP kinase module (MAPKKK, MAPKK, MAPK) downstream of activators (GTPases, and Src in the case of ERK5) connected to tyrosine kinase receptors, cytokines receptors and stress sensors. The signals transited sequentially through these steps converging on the activation of different cytoplasmic and nuclear effectors that trigger appropriate cellular responses.

The MAPK cascades are highly conserved signaling networks that transduce the signals elicited by physiological and stress stimuli [1–4] and (Figure 1). The ERK pathway is the best known of these cascades. It is activated typically through the binding of agonists to tyrosine kinase (TK) receptors, which results in auto-phosphorylation of tyrosyl residues on the receptors, hence creating docking sites for adapter proteins and enzymes. Then, follows the activation in cascade of the GTPase Ras, the MAP kinase kinase kinase (MAPKKK) Raf, the MAP kinase kinases (MAPKK) MEK-1/2 and finally the MAP kinases (MAPK) ERK1/2. In turn, ERK phosphorylates a number of cytoplasmic and nuclear proteins [4]. The functions of the signaling molecules along the MAPK pathways are regulated by scaffolding proteins such as CNK in the ERK pathway [5, 6]. The scaffolding proteins facilitate or restrict the enzyme/substrate interactions by modulating the availability of the signaling components [6–8]. They may also contribute to proper signal dissemination by directing the cascade to specific upstream receptors or to unique downstream targets. Moreover, they contribute to stabilize signaling components, determine the signaling thresholds, protect signaling components from phosphatases or direct the localization of the cascade components [7, 9]. Along these lines, β-arrestin scaffolds the Raf-MEK-ERK pathway and it increases angiotensin II-induced Raf and MEK activation and targeting of activated ERK2 to endosomes [7, 9]. The interaction of β-arrestin with activated ERK1/2 is irreversible and allows these kinases to phosphorylate cytoplasmic substrates without major effects on nuclear targets [10]. The ERK cascade is mostly considered as a mitogenic and survival pathway following its activation by growth factors. ERK activation is also induced by stress including ROS or ROS-generating agents [3, 11].

Studies in the field of “stressbiology” have revealed the existence of other MAPK cascades that are activated more specifically by stresses. These members of the MAPK family are called stress-activated protein kinases (SAPKs). The best-characterized SAPKs are members of the JNK and p38 families [12, 13]. ERK5, another member of the MAP kinase family, also responds to stress stimuli, especially shear stress and is sometimes considered as a SAPK [14–16]. As for ERK, the induction of the SAPK pathways involves the activation in cascade of small GTPases, upstream of MAPKKK and MAKK [13]. While there is a large degree of specificity within the different MAPK cascades, there is also significant overlap between them. Both upstream activators and downstream targets can be shared between different subfamilies, allowing cross-talk and feedback signaling events between MAPK family members [15]. Notably, both ERK and p38 MAP kinase pathways are activated by VEGF (Vascular Endothelial Growth Factor) downstream of VEGFR2 [17–19]. Moreover, both p38 and JNK share downstream effectors such as ATF2 [20].

The p38 pathway is a major signaling pathway in the endothelial compartment as p38 plays central roles in regulating endothelial cell functions in response to oxidative stress and during cancer progression and metastasis. Accordingly, this review will focus on the biological functions regulated by p38 in the endothelial compartment.

## THE P38 PATHWAYS

The p38 MAP kinases consist of four isoforms (α, β, γ and δ) that are encoded by four different genes that share a high sequence homology [13, 21]. p38α/MAPK14 is ubiquitously expressed, whereas the other isoforms are preferentially expressed in certain tissues. Notably, p38β is mostly found in the brain, whereas p38γ is significantly expressed in skeletal muscles and p38δ is mainly found in the pancreas, testis, kidneys and small intestine [13]. p38α is the isoform that is the best characterized and mostly studied and will be referred as p38 in this review. There is a certain level of redundancy in the functions of the four isoforms [22]. Nevertheless, if mice knockout for p38β, p38γ and p38δ isoforms have a normal development, the knockout of p38α is embryonically lethal, highlighting the point that p38α has specific essential functions [23].

p38α was first identified in mammalian cells as a polypeptide of 38 kDa that is tyrosine phosphorylated in response to endotoxin and hyper-osmotic shock [24]. Almost concomitantly, it was shown as the activator of MAPKAP-K2 (MAP kinase-activated protein kinase-2: MK2) [25], a kinase involved in phosphorylation of the small heat shock protein HSP27 in response to various stimuli including oxidative stress, VEGF and IL-1β [26–29]. During the same period, the group of JC. Lee identified p38α as the main target of a class of imidazole pyridinyl anti-inflammatory drugs that include SB203580 [30]. These findings paved the roads to fascinating discoveries during the last 20 years.

### Activation of p38

As for the other MAP kinases (Figure 1), the p38 pathway involves the canonical activation in cascade of small GTPases, namely Rac or Cdc42, upstream of the MAP kinase module. This latter consists of MAP Kinase Kinase Kinases (MAPKKKs, MKKKs or MEKKs), MAP Kinase Kinases (MAPKKs, MKKs) and p38 itself [13, 18]. Among MKKKs activating p38, there are MEKK3/4, TAK1 (TGFβ-activated kinase 1) and ASK1 [22]. In response to oxidative stress, ASK1 is the major MKKK that induces the activation of the p38 pathway. The reduced form of the antioxidant protein thioredoxin (TRX) plays a crucial role in ASK1 activation by ROS. Notably, TRX interacts with ASK1 at its N-terminus, preventing its oligomerization and therefore its activation. ROS induce the oxidation of TRX, which then dissociates from ASK1 allowing its oligomerization and enabling its autophosphorylation and activation. In several types of cells, activated ASK1 induces the activation of the p38 cascade, leading to a pro-apoptotic signal [31, 32]. Very interestingly, ceramide, a bioactive sphingolipid located in the plasma membrane, is implicated in ASK1-mediated stress response, upstream of p38 in both keratinocytes [33] and lymphoid cells [34]. Along these lines, ceramide participates to ASK1 phosphorylation in endothelial cells exposed to radiation-induced oxidative stress [35].

**Figure 2:**
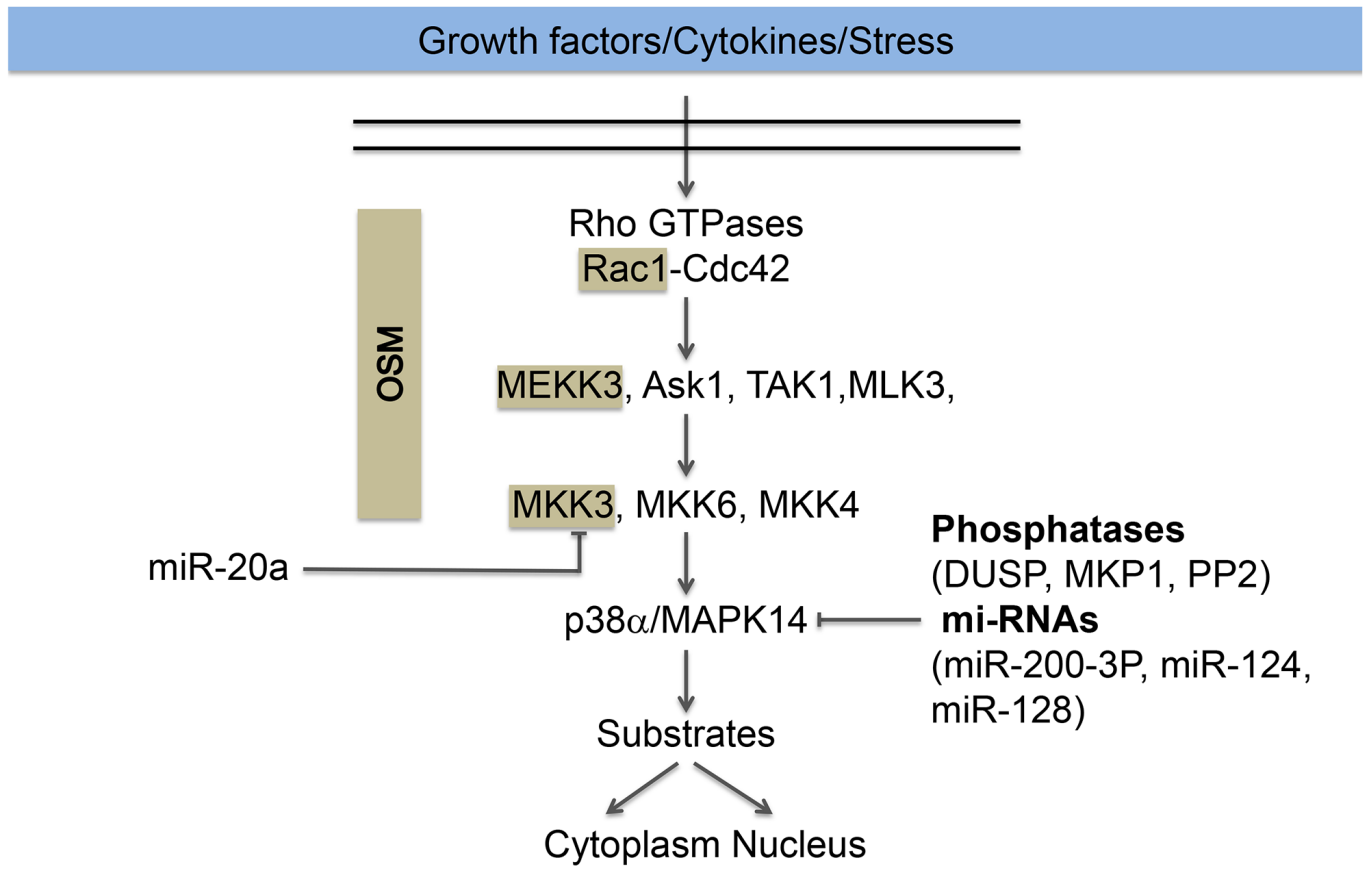
Regulation of the p38 pathway The p38 pathway is activated in response to growth factors (VEGF), cytokines (TNFα) and stress (oxidative stress). Its activation typically involved the activation of the small GTPases Rac1 or Cdc42, upstream of the MAP kinase module MAPKKK (ASK1, TAK1, MEKK3, MLK3), MAPKK (MKK3/6/4) and the MAP kinase p38α/MAP14, herein named p38. Activation of p38 converges on the regulation of nuclear and cytoplasmic substrates described in the text. The cascade Rac, MEKK3, and MKK3 for the activation of p38 MAPK is maintained together and targeted to membranes ruffles by the scaffolding protein OSM (Osmosensing Scaffold for MEKK3). The scaffolded proteins are highlighted in beige. Dephosphorylation of the activation sites by phosphatases (DUSP, MKP1, PP2) or repression of p38 expression by miR-200-3-P, miR-124 and miR-128 contributes to p38 inactivation. On the other hand, miR20a repress the expression of MKK3, which also impairs the activation of p38.

Activated MKKKs induce the activation of MKKs by their phosphorylation at both serine and threonine residues located in the activation loop. The MKKs mainly involved in p38 activation are MKK3 and MKK6 [36, 37]. *In vitro*, MKK4 can also phosphorylate p38 [38]. However, MKK4 is preferably an activator of JNK and MKK3 and MKK6 are the predominant activator of the p38 pathway [39]. These latter activate p38 in a conventional manner by dual phosphorylation at Thr180 and Tyr182 residues at a Thr-Gly-Tyr motif [37] (Figure 2). The resulting conformational change within p38 enhances its kinase activity by a subsequent increase in the accessibility of the substrate at the catalytic site. MKK3/6 appear to have redundant functions during development, as MKK3 or MKK6 knockout mice are viable and healthy whereas the double knockout mice died in mid-gestation with defects in the placenta and embryonic vasculature [13, 39]. The biological effects resulting from p38 activation depend on both the duration and magnitude of the signal. Therefore, inactivation of p38 is a crucial mechanism regulating its biological-activated functions.

Intriguingly, there are non-canonical mechanisms of p38 activation. For example, in T cells, p38 may be activated via TCR-mediated-p38 phosphorylation at Tyr323 by the ZAP70 protein kinase (ζ-chain-associated protein kinase of 70 kDa) [40]. Additionally, the binding of TAB1 (TAK1-binding protein 1) induces a conformational rearrangement of p38 in its activation loop enabling autophosphorylation of p38 [41, 42]. These non-canonical mechanisms of p38 activation still remain poorly characterized.

The p38 module is kept together by scaffolding proteins that play a major role in the specificity of its signaling. These proteins are devoid of intrinsic catalytic activity but they are involved in regulating the specificity of the p38 cascade by enabling interaction between the cascade members, by regulating the conformational changes necessary for their activation and by securing their cellular localization [22, 43] (Figure 2). Notably, OSM (Osmosensing Scaffold for MEKK3) interacts with Rac, MEKK3 and MKK3, forming a protein complex that is required for p38 activation in response to osmotic shock in mammalian cells [43]. Moreover, OSM also targets the p38 module to membrane ruffles [44]. On the other hand, RACK1 (Receptor for Activated C Kinase 1) modulates the activation of the p38 pathway by interacting directly with MKK3/6 and increasing their kinase activity without affecting their phosphorylation. The enhanced p38 activation mediated by RACK1 suppressed TNFα-induced cell death in L929 cells [45]. Intriguingly, this latter study further shows that the endogenous scaffolding interaction between MKK3/6 is increased upon stimulation by TNFα, which suggests that the phosphorylation of MKK3/6 is important for their interaction with RACK1. In corollary, it is suggested that a yet unidentified conformational change results from MKK3/6 phosphorylation and leads to the subsequent binding to RACK1 [45]. Hence, it seems that RACK1 may act both as a scaffolding protein and as an allosteric modulator to underlie its interaction with MKK3/6. Of note, JIP2 (JNK-interacting proteins-2) can regulate signaling of the p38 pathway by interacting directly with p38, MKK3 and regulators of Rac [46].

### Inactivation of p38

Signaling specificity is a major issue in the cellular response to extracellular and intracellular stimuli. Emerging evidence indicate that it is tightly regulated by the timing and intensity of the activation. In that regard, the inactivation of p38 to rapidly shut-off its activity and to allow a transient activation is a major contributing factor underlying the specificity of the cellular response [22]. Dephosphorylation of p38 and reduced expression by miRNA are major mechanisms that contribute to p38 inactivation (Figure 2).

#### Phosphatases

p38 is activated by dual phosphorylation at Thr180 and Tyr182 residues at a Thr-Gly-Tyr motif located in the activation loop [37]. Accordingly, a quick way to shut-off the activation of p38 is to dephosphorylate these sites. This is achieved by phosphatases, which may also act upstream to dephosphorylate MKKs. Notably, members of the phosphatase family DUSPs (Dual-Specificity Phosphatases), including the MKPs (MAPK phosphatases), can deactivate p38 by dephosphorylating both Thr180 and Tyr182 within p38 [47]. The interaction of DUSP with MAPK changes the conformation of the enzyme, which increases its phosphatase activity. Interestingly, these phosphatases can be activated by various stimuli including those that activate p38 [48]. Dephosphorylation of p38 by DUSP is especially important in a cardiovascular context given that p38 is overexpressed in several cardiovascular diseases associated with oxidative stress, including myocardial infarction, heart hypertrophy, heart failure and ischemic heart diseases [49].

Members of the PP2 family of serine threonine phosphatases are other phosphatases reported to inactivate p38. For example, PP2C and Wip1 (Wild-type p53-induced phosphatase) inactivate p38 indirectly by targeting MKKS upstream of p38 or directly by shutting-off p38 by dephosphorylation of Thr180 residue [50, 51]. In addition, PP2A can interact directly with the active form of p38 to deactivate the kinase. In that regard, its inhibition maintains the phosphorylated state of p38 [52]. In different types of tumor cells [53–55] and macrophages [55], the inhibition of PP2A is associated with an increase in p38 activity in response to diverse stimuli. Yet, the role of PP2a in deactivation of p38 remains cell type- and stimuli-dependent. Indeed, inhibition of PP2A in endothelial cells is associated with increased phosphorylation of p38 in unstimulated cells but does not affect oxidative stress-induced p38 activation [56]. Interestingly, in endothelial cells, MAP kinase phosphatase-1 (MKP-1) is also a negative regulator of p38 activity and p38-dependant VCAM expression in response to shear stress [57]. Recently, R. Singh proposed a model that predicts that MKP1 and TAB1 regulate p38 initial transient activation and its basal activity through positive and negative feedback loops in response to IL-1 [58].

#### MicroRNAs

MicroRNAs (miRNAs) are a conserved class of small non-coding RNAs that direct post-transcriptional silencing of complementary mRNA targets following assembling with Argonaute proteins into miRNA-induced silencing complexes (miRISCs) [59]. Several lines of evidence indicate that miRNAs contribute to the homeostasic signaling of the p38 pathway in response to various activators. Notably, the miR-17 ˜ 92 cluster is a global regulator of the ASK1 signalosome, which is a central actor in the p38-mediated inflammatory process activated during rheumatoid arthritis [60]. Along these lines, miR-20a, a member of the miR-17 ˜ 92 cluster, represses the activity of p38 induced by VEGF by directly targeting MKK3 mRNA [61]. Furthermore, miR-196a repressed p38-dependant cell migration in response to VEGF by binding to the 3’UTR region of Annexin A1 mRNA [62]. In neurons, miR-124 and -128 deplete p38 and repress the p38-dependent translation machinery [63]. During lung development, mir-17 family of miRNA modulates FGF10-FGFR2b downstream signaling by specifically targeting Stat3 and p38. In turn, this contributes to regulate E-cadherin expression, which modulates epithelial bud morphogenesis in response to FGF10 signaling [64]. Interestingly, a cross-talk involving p38 has been reported between members of the miR-200 family and oxidative stress. Notably, the members of the miR-200 family are markedly enhanced in a p53-dependent manner in hepatic cells by H_2_O_2_ treatment. This upregulation of miR-200-3p in turn modulates the H_2_O_2_-mediated oxidative stress response by targeting and repressing p38. The enhanced expression of miR-200-3p mimics p38 deficiency and promotes H_2_O_2_-induced cell death. It is concluded that the p53-dependent expression of miR-200a-3p promotes cell death by inhibiting a p38/p53/miR-200 feedback loop [65]. Based on these findings, one can expect in the coming years that microRNA will reveal to be major regulators underlying the specificity and long-term sensitivity of not only p38 but also of other signaling pathways.

### Sub-cellular localization and substrates of p38

#### Sub-cellular localization

p38 resides both in the nucleus and the cytoplasm and its cellular localization is an important determinant of its signaling specificity [66, 67]. In resting cells, p38 is mostly found in the cytoplasm in its non-phosphorylated inactive state. Along these lines, impairing dephosphorylation of p38 with a catalytically inactive mutant of MKP-1 (MKP-1/CS) traps p38 in the cytoplasm in response to endothelin-1 in rat mesangial cells [68]. Following activation, a pool of phosphorylated p38 remains in the cytoplasm to regulate the functions of cytoplasmic proteins. However, depending on the cell type, the nature of the stimulus and even the age of the cells, another pool of phosphorylated p38 may redistribute to the nucleus, which enables its access to particular nuclear substrates [22, 69–71]. For example, nuclear transport of p38 is a key aspect of the cellular response to UV irradiation [71]. Intriguingly, p38 may also be present in the nucleus of resting cells and can be exported to the cytoplasm [66].

Only few studies aim at understanding the mechanisms involved in the nucleo-cytoplasmic translocation of p38. In response to inducers of DNA damage, the nuclear localization of p38 is dependent on its phosphorylation, but not on its kinase activity [22, 72]. Given that p38 has no NLS sequence, its nuclear translocation requires the intervention of partner proteins such as importins or proteins having an NLS sequence [22]. Recently, it has been shown that p38 translocation involves three beta-like importins 3,7 and 9 [73]. On the other hand, the nuclear export of p38 requires its dephosphorylation and is dependent on MK2 but not on a nuclear export signal (NES) [74].

Depending on its location, p38 can regulate several cellular functions by acting on both cytoplasmic and nuclear substrates. Notably, p38 directly phosphorylates close to 100 proteins that include protein kinases and transcription factors. We will only briefly mention some of them, as this issue has been reviewed in details by Angel Nebreda et al. [75, 76].

#### Nuclear substrates of p38

About 55% of p38 substrates are located in the nucleus. Most of them are DNA and RNA binding proteins that regulate gene expression. Notably, p38 directly phosphorylates at least 31 transcription factors, which most of the time triggers transcriptional activation [75, 76]. CHOP (GADD153) is one of the first transcription factor reported to be activated by p38. Its activation by p38 following ROS-induced DNA damage leads to cell cycle arrest at the G1/S boundary, allowing DNA repair [77]. ATF2 (cyclic AMP-dependent Transcription Factor-2), p53, STAT-3 (Signal Transducer and Activator of Transcription-3), are other transcription factors activated by p38 [32, 78–80]. Their p38-mediated phosphorylation triggers the transcription of genes involved in the regulation of apoptosis, cell growth, immune response and response to DNA damage. For example, the phosphorylation of p53 by p38 regulates the G2/M transition [81, 82] On the other hand, HBP1 (HMG-box transcription factor 1) is a p38 target that represses cell cycle progression at G1 [83]. Interestingly, in endothelial cells, p38 controls the transcriptional activation of NFκB and the production of TNFα via phosphorylation of RelA in response to *Borrelia burgdoferi* antigens [84]. Moreover, we recently found that activation of endothelial p38 by IL-1β regulates the transcription of miR-31 by activation of c-fos and GATA2 [85]. In turn, miR-31 represses the expression of E-selectin and thereby adhesion and transendothelial migration of colon cancer cells [85]. Intriguingly, another study indicates that p38 supports the nuclear functions of estrogen receptor by contributing to its phosphorylation [86].

Many transcription factors are not direct target of p38 but are targeted by downstream substrates of p38 such as MK2 and its substrates Cdc25b and Hur [75, 76]. The transcription factor CREB is also phosphorylated by MK2 and by other p38 substrates such as mitogen and stress-activated protein kinase 1/2 (MSK1 and MSK2) [87, 88]. MSK1 and MSK2 also phosphorylate ATF1 and histone H3. Additionally, MAP kinase-interacting serine/threonine-protein kinases 1 and 2 (MNK1 and MNK2) phosphorylate the initiation factor eIFAE, which regulates protein synthesis [89]. Intriguingly, some proteins can be phosphorylated by both p38 and MK2. This double targeting of substrates might function as fine-tuning mechanisms to prevent inappropriate activation of effectors [75, 76].

Of note, p38 is also connected to chromatin remodeling by phosphorylating BAF60c and p18^Hamlet^, two structural constituents of SWI/SNF and SCRAP complexes, respectively [75]. Additionnaly, FBP2/3 and SPF45 are p38 substrates that regulate mRNA processing whereas HuR and KSRP regulate mRNA stability [90]. On the other hand, MK2 and MK3 regulate mRNA stability by phosphorylating ARE-binding proteins such as TTP or HuR [91].

In summary, p38 pathway regulates repressors or activators of transcription as well as chromatin remodeling, enabling or not the transcription of many genes involved in various cellular processes [22, 92].

#### Cytosolic substrates of p38

Many cytoplasmic proteins are phosphorylated by p38 or its effector kinases. These substrates include proteins that mediate the anti-proliferative functions of p38 such as p57Kip2 and cyclin D1/3 [93, 94], and apoptosis: Bax and BimEL [95]. p38 also regulates cell survival through the phosphorylation of caspase-3 and caspase-8 [96]. It modulates the turnover of proteins by inducing phosphorylation-mediated changes in substrates stability or by phosphorylating Siah2, a ring finger E3 ligase [ 97]. On the other hand, p38 inhibits proteasome activity in response to hyperosmotic shock by phosphorylating the proteasome regulatory subunit Rpn2 [98]. Activated p38 also phosphorylates EGF receptor to promote its internalization [22]. As discussed below, by contributing to the phosphorylation of heat-shock protein 27 (HSP27) and annexin A1 (ANXA1), the p38 pathway mediates actin-based motility by regulating actin remodeling and cell contractility in response to VEGF in endothelial cells [28, 99, 100].

## THE P38 PATHWAY AS A MAJOR REGULATOR OF THE OXIDATIVE STRESS RESPONSE IN ENDOTHELIAL CELLS

### Reactive oxygen species and oxidative stress

Reactive oxygen species (ROS) are produced from molecular oxygen O_2_. Oxygen is unreactive in its ground state but is reduced to water under normal metabolic conditions. This occurs via a stepwise pathway during which partially reduced and very reactive intermediates are produced. These reactive intermediates have a strong oxidizing potential and a low half-life. These ROS include the superoxide radical (O_2_^.-^), hydrogen peroxide (H_2_O_2_) and the hydroxyl radical (OH.) that is the most reactive of them [101]. Reactive nitrogen species (RNS) are other ROS intermediates that are derived from nitrogen metabolism. They are mainly NO and its derivatives: nitrogen dioxide (NO_2_) and peroxynitrite (ONOO-). Notably, NO is synthesized by the enzyme NOS (nitric oxide synthase) and it interacts with O_2_^.-^ to form peroxynitrite ONOO-, a very reactive compound that reacts with many molecules, via a process called nitrosylation [102].

### Reactive oxygen species and oxidative stress in the endothelial compartment

**Figure 3:**
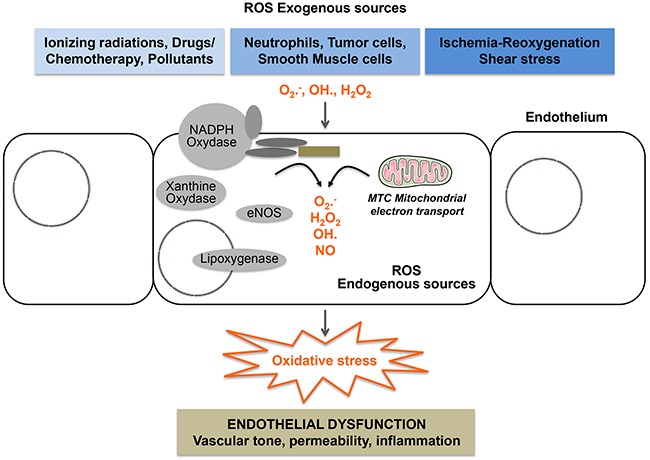
Endothelial cells are heavily exposed to oxidative stress All components of the vascular wall including endothelial cells produce ROS from endogenous sources. Enzymatic sources include the NADPH oxydase complexes, lipoxygenases, xanthine oxidase, uncoupled eNOS, uncoupled mitochondrial electron transport. They are also exposed to exogenous sources of ROS generated by drugs, ionizing radiation, ischemia/reoxygenation, shear stress and circulating cells including neutrophils and cancer cells.

Endothelial cells are heavily exposed to ROS and these latter are major regulators of physiological and pathological processes involving the endothelium. Notably, endothelial cells are exposed to both endogenous and exogenous sources of ROS (Figure 3). Endogenous ROS are mainly produced by the mitochondrial respiratory chain and also by enzymatic reactions involving NADPH oxidase (NOX), xanthine oxidase, nitric oxide synthase (NOS), arachidonic acid, and metabolizing enzymes such as the cytochrome P450 enzymes, lipoxygenase, and cyclooxygenase [103]. Exogenous sources of ROS are generated mainly by ionizing radiation, UV, xenobiotics and pollutants [56].

Endothelial cells are also exposed to ROS generated by their surrounding cells such as smooth muscle cells and to ROS originating from the blood. Notably, ROS may originate from circulating blood leukocytes, activated phagocytes during the respiratory burst, oxidizing xenobiotics and also from shear stress [104–106]. Endothelial cells are themselves generators of ROS [105, 107, 108]. In response to TNFα and angiotensin-II, these cells produce H_2_O_2_ that acts as a second messenger triggering endothelial cell activation. Endothelial cells are also equipped with eNOS, a specific nitric oxide synthase, that produces NO, which in turn transduces responses generated by histamine and VEGF [109, 110]. Moreover, endothelial cells produce ROS in response to ischemia-reoxygenation, which contributes to the modulation of intracellular calcium levels and calcium-dependent signaling [111, 112]. Additionally, ROS are by-products of the activation of cyclo-oxygenases by bradykinin in endothelial cells [113]. ROS are also second messengers of the signals generated by stresses, particularly shear stress [107, 114]. Interestingly, several signals induced by diverse agonists including Fas-L, endostatin, TNFα, H_2_O_2_ and homocystein stimulate the formation of lipid rafts redox signaling platforms in the plasma membrane of endothelial cells [115]. These signaling platforms provide important driving forces in order to activate the NADPH oxidase complex, therefore producing superoxide anion radical and generating intracellular oxidative stress.

Increased levels of ROS due to an imbalanced regulation of the oxidation/reduction (redox) metabolism create a condition known as oxidative stress. This leads to a variety of biochemical and physiological lesions underlying metabolic impairment, cancer initiation and progression and ultimately cell death. Several types of antioxidants regulate ROS homeostasis. This includes dietary natural antioxidants (vitamins A, C, and E), endogenous antioxidant enzymes (superoxide dismutase, catalase, glutathione peroxidase, glutathione reductase, and peroxiredoxins), and other antioxidant molecules (glutathione, coenzyme Q, ferritin, and bilirubin).

### Endothelial response to oxidative stress: impact on endothelial homeostasis and dysfunction

In reason of the heavy exposure of endothelial cells to ROS, it is not surprising that oxyradicals profoundly affect the functions of these cells and thereby the cardiovascular homeostasis by contributing, for example, to the modulation of NO generation and thereby to the vascular tone [106, 116, 117]. On the other hand, the inability of endothelial cells to cope with acute or chronic exposure to oxidative stress is associated with a loss of endothelial integrity leading to endothelial dysfunction. Endothelial dysfunction is characterized by a reduction of the bioavailability of vasodilator agents such as NO that is concomitant with an increase in endothelium-derived contracting factors [118, 119]. This imbalance results in an impairment of endothelium-dependent vasodilation, the basic functional characteristic of endothelial dysfunction. By extension, endothelial dysfunction also refers to any deregulated mechanism that affects the normal functions of the endothelium such as maintaining the integrity of the endothelial barrier. The impaired endothelium-dependent functioning is associated with specific state of endothelial activation, which is characterized by a pro-inflammatory, proliferative, and pro-thrombotic states that favor all stages of atherogenesis [119]. The physiopathological status of an individual endothelial function may reflect the propensity to develop atherosclerotic and other endothelial dysfunction-related diseases including cancer as discussed below.

Hence, it is of high clinical importance to better understand how p38 regulates endothelial cell response to ROS in normal and pathological conditions.

### p38 regulates cytoskeletal dynamics in response to oxidative stress

#### p38 regulates actin remodeling as an early adaptive response to oxidative stress

**Figure 4:**
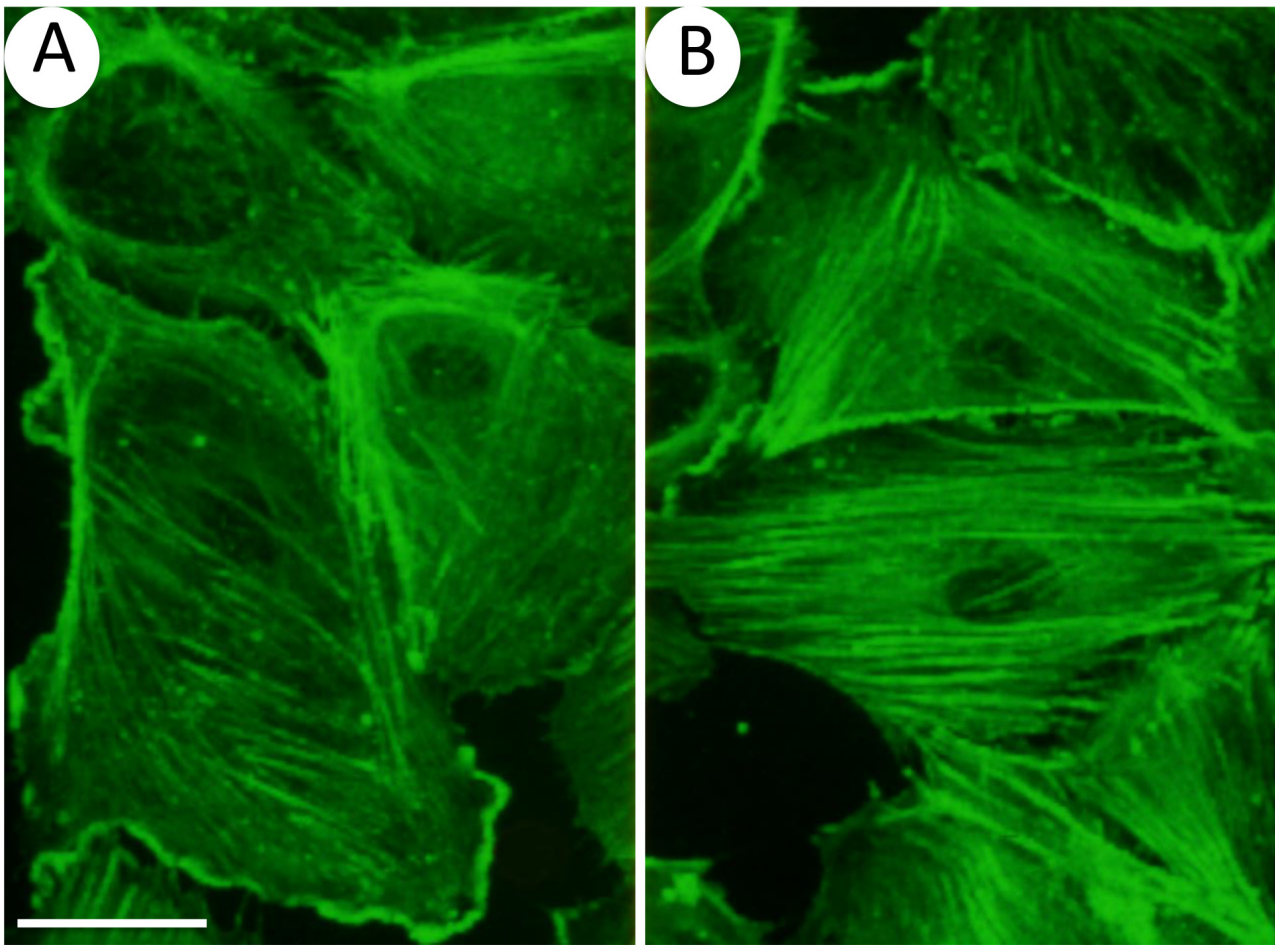
Oxidative stress induces actin remodeling in endothelial cells Endothelial cells (HUVECs) plated on gelatin-coated petri dishes were left untreated **(A)** or were exposed 30 minutes to 50 μM H_2_O_2_ **(B)** F-actin was stained using FITC-conjugated phalloidin and cells were examined by confocal microscopy. Results show that H_2_O_2_ induces a massive remodeling of actin into stress fibers. 60 x and bar = 25 μm.

In various types of cells, including hepatocytes and fibroblasts, oxidative stress produces a severe disruption of actin cytoskeleton characterized by fragmentation and patching of F-actin [120, 121]. In contrast, in endothelial cells exposed to ROS in concentration as low as 50 μM, a major and early response of endothelial cells is characterized by actin remodeling into stress fibers [27] and (Figure 4A, 4B). Several lines of evidence support the point that the p38/MK2/HSP27 pathway is importantly involved in transducing this effect. Notably, inhibiting p38 activity with SB203580 abrogates both activation of MK2 and phosphorylation of the actin polymerizing factor HSP27, which is associated with an inhibition of H_2_O_2_-induced actin remodeling in stress fibers [27]. The role of HSP27 phosphorylation in mediating microfilament reorganization in response to ROS is supported by the fact that transfected fibroblasts that express the same amount of HSP27 as endothelial cells respond similarly to endothelial cells following phosphorylation of HSP27 by H_2_O_2_ [27, 120]. Moreover, the H_2_O_2_-mediated actin remodeling does not occur in high HSP27-expressing fibroblasts treated with SB203580 or expressing a non-phosphorylatable mutant of HSP27. Hence, HSP27 phosphorylation downstream of p38 constitutes a quick adaptive and protective function against ROS-induced cytoskeletal toxicity in endothelial cells. Along these lines, HSP27 phosphorylation triggers a protective response against serum obtained from burn patients, inducing endothelial barrier dysfunction [122]. Interestingly, the p38-mediated actin remodeling into stress fibers in response to ROS occurs within 5 min [27], suggesting that it represents a very early adaptive response of endothelial cells to ROS [27]. Accordingly, a high amount of stress fibers is observed in endothelial cells exposed to shear stress suggesting that stress fibers contribute to maintain the integrity of the endothelium by enhancing adhesion to the substratum [123]. On the other hand, the p38/MK2/HSP27 axis-mediated actin remodeling might play a role in the regulation of endothelial barrier, as it will be discussed below. p38-mediated actin remodeling is also compatible with the role of ROS in promoting migration of endothelial cell [99, 124].

Interestingly, in endothelial cells, ERK is stimulated concomitantly to p38 in response to ROS, which suggests that multiple parallel MAPK pathways may be important in endothelial cells and cardiovascular response to cellular stresses [125, 126]. Along these lines, the strong actin polymerizing activity generated through the p38 pathway following exposure to ROS leads to early membrane blebbing and late manifestations of apoptosis under conditions in which ERK activity is not concomitantly induced and in which focal adhesions are misassembled [27, 127, 128]. Moreover, ERK activation by ROS is required to mediate DAPK (Death-Associated Protein Kinase-1) -induced phosphorylation of tropomyosin-1 α chain (TM1) at Ser283 enabling elongation of actin filaments to increase endothelial cell contractility and assembly of focal adhesions [129]. It should be noted that the myosin module is another important component of stress fibers [130]. In fact, phosphorylation of myosin light chain (MLC) promotes the productive interaction of myosin heads with actin filaments, thus stimulating myosin ATPase activity and generating contractility. In turn, increased myosin-actin contractility results in bundling of F-actin into thick stress fibers and triggers the assembly of focal adhesions [130]. Importantly, the phosphorylation of TM1 might contribute to increase the activity of MLC ATPase, thereby increasing the association of actin with myosin and leading subsequently to formation of stress fibers and cell contractility [130, 131]. Overall, these findings suggest that the ERK/TM1-dependent assembly of focal adhesions, in concert with p38-mediated increase in actin polymerization, is a major regulator of the endothelial cell functions that depend on actin remodeling [132]. Additionally, a loss of this coordinate activation of these pathways in response to ROS results in a breaking of the endothelium integrity. In this context, the loss of TM1 phosphorylation following blocking of the ERK pathway is associated with an increased in endothelial permeability and trans-endothelial migration of colon cancer cells [129, 132, 133]. Overall, we hypothesize that activation of ERK/TM1 participate with p38 to the quick adaptive response of endothelial cells to ROS.

#### p38 regulates endothelial permeability in response to oxidative stress

The vascular endothelium is at the interface between the blood and the interstitial space of all organs. It acts as a semi-permeable barrier between these two compartments and participates to the regulation of macromolecules transport and blood cells trafficking through the vessel wall. Under physiological conditions, the permeability of the endothelium is tightly regulated, and the maintenance of its size-selective sieving properties is critical for several physiological functions, including normaltissues homeostasis, vessel tone, host defense and angiogenesis [134]. Nevertheless, as seen below, deregulation of endothelial permeability is also associated with endothelial dysfunctions. Two complementary pathways underlie endothelial permeability, namely transcellular and paracellular pathways. Under basal conditions, the transcellular pathway mediates the transport of plasma proteins such as albumin whereas smaller molecules such as glucose are transported via the para- or inter-endothelial pathway [135]. On the other hand, most of the permeability-increasing mediators such as histamine, VEGF and PAF (platelet activating factor) increase endothelial permeability via the paracellular pathway by opening inter-endothelial junctions (IEJs). This involves alteration of the balance between competing contractile forces and cell-cell and cell-extracellular matrix (ECM) adhesive forces [136]. Actin cytoskeleton rearrangement and actinomyosin interaction are centrally involved in regulating IEJ. Notably, they are involved in both endothelial cell retraction and gap formation [137, 138]. Along these lines, remodeling of the endothelial actin cytoskeleton, such as cortical actin dissolution and increase in actin stress fiber density, leads to cell contraction and alteration in cell shape, providing a structural basis for increase of endothelial cell permeability. In that regard, several studies have shown that the p38 pathway along with the RhoA/ROCK pathways are important regulators of these cytoskeletal alterations underlying paracellular endothelial permeability [134, 136]. In the case of p38, its activation by thrombin and histamine regulates increased endothelial permeability via actin remodeling into stress fibers, which will contribute to increase retracting forces and open IEJs [136, 139]. In turn, this will be associated with the inflammatory process and may lead to endothelial dysfunction [136]. Interestingly, oxidative stress is a major inducer of endothelial permeability following actin remodeling into stress fibers associated with the phosphorylation of MARCKS (actin-binding protein myristoylated alanine-rich C-kinase substrate) [140]. Along the same lines, exposure of lung endothelial cells to more than 1 hour to soluble components of cigarette smoke-generating ROS have direct disruptive effects on endothelial barrier that could be counteracted by inhibitors of p38 [141]. In this context, we have found that activation of the p38/MK2/HSP27 axis protects endothelial cell from ROS-induced fragmentation of F-actin via reorganization of the actin cytoskeleton in stress fibers [120], which in turn may contribute to the regulation of the endothelial barrier. Overall, these findings are consistent with the fact that the p38/MK2/HSP27 axis contributes to regulate endothelial permeability to ROS.

### p38 regulates DNA damage, cell death and senescence in response to oxidative stress

#### p38/nucleophosmin and DNA damage response

Over the last decade, the role of the p38 pathway as an important part of the DNA damage signaling response has been highlighted. DNA damage-inducing agents (UV, IR, doxorubicin, cisplatin) are known activators of p38 MAPK. p38 activation contributes to the regulation of cell cycle checkpoints G2/M and to a lesser extent G1/S. p38 activates the p53/GADD45α/14.3.3 pathway leading to inhibition of Cdc2/cyclinB but can also inhibit the phosphatase Cdc25B via MK2, impairing in both cases the G2/M transition. The role of p38 in G1/S crossing is less well established but it implies p53, regulation of cyclin D1 and/or phosphorylation and degradation of Cdc25A [82].

**Figure 5:**
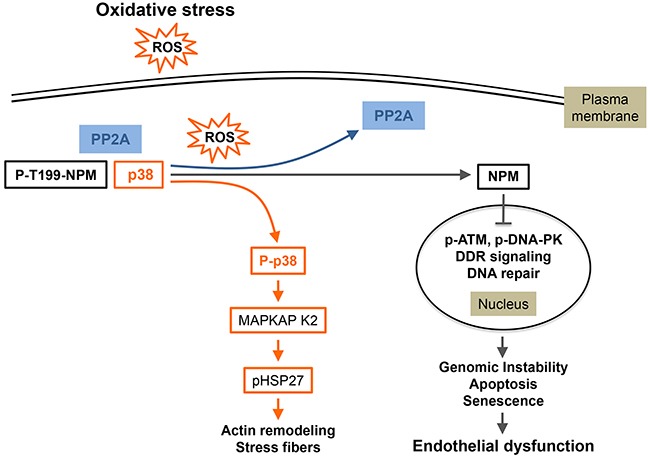
p38-mediated oxidative stress responses in endothelial cells In unstimulated quiescent endothelial cells, p38/p-T199NPM/PP2a form a complex that is quickly dissociated in response to oxidative stress (ROS). This dissociation happens concomitantly with activated PP2a-mediated dephosphorylation of NPM at T199. Following dissociation of the complex, p38 phosphorylated in response to ROS exposure activates its downstream effectors notably HSP27 leading to actin reorganization in conjunction with the activation of the ERK/DAPK1/Tropomyosin 1α chain axis (see text). Moreover, a pool of NPM unphosphorylated at T199 translocates to the nucleus where it is associated with an impaired DDR due to impaired detection of DNA damage, as reflected by the inhibition of DNA-PK and ATM/ChK2. This may lead to genomic instability, apoptosis, senescence and thereby endothelial dysfunction. (Adapted from 56).

Recently, we characterized in endothelial cells a new mechanism of DNA damage response implying a novel p38 partner that we identified, by a proteomic approach, as nucleophosmin (NPM) [56]. Co-immunoprecipitation and microscopic analysis using Proximity Ligation Assay confirm the existence of a cytosolic NPM/p38 interaction in basal condition. Oxidative stress, generated by a short exposure to 250 or 500 μM of H_2_O_2_, induces dephosphorylation of NPM at T199 that depends on phosphatase PP2a, another partner of the NPM/p38 complex. Blocking PP2a activity or knocking down its expression lead to accumulation of NPM-pT199 and to an increased association of NPM with p38 without affecting oxidative stress-induced activation of p38. Concomitantly to NPM dephosphorylation, oxidative stress promotes the translocation of this protein to the nucleus to modulate DNA damage response (DDR). Notably, dephosphorylated NPM at T199 impairs the phosphorylation of DNA double-strand breaks (DSBs)-sensing protein DNA-dependent protein kinase (DNA-PK) downstream of ATM and subsequent formation of γ-H2AX foci [56]. Moreover, the non-phosphorylatable NPM/T199A mutant delayed the DSB repair as revealed by comet assays, leaving cells with unrepaired DSB. The establishment of long-lasting unrepaired DNA damage may be associated with genomic instability leading ultimately to death, growth arrest or senescence and would contribute to oxidative stress-induced endothelial dysfunction [56]. Overall, these results suggest that the p38/NPM/PP2a complex acts as a dynamic sensor allowing endothelial cells to initiate nuclear response to acute and massive oxidative stress and to translate these molecular events into late functional effects [56]. In contrast, lower concentration of H_2_O_2_ (50μM) does not trigger the dissociation of NPM from p38, which is consistent with the fact that at this more physiological concentration the p38/NPM complex may induce a protective function against ROS [56]. The biological issues covered by these findings highlight new mechanisms helping to understand oxidative stress-induced endothelial dysfunction-associated disorders that include normal tissue toxicity during radiotherapy and pathologies such as atherosclerosis and diabetes. In the case of diabetes, the link between oxidative stress and endothelial dysfunction is based on the fact that hyperglycemia generates ROS via various signaling pathways. In turn ROS induced endothelial dysfunction that is associated with diabetic complication including atherosclerosis and diabetic retinopathy [142]. On the other hand, it is possible that a pool of p38 released from NPM following oxidative stress becomes activated and together with ERK activation becomes responsible for the actin remodeling into stress fibers as described in the preceding section. Taken together, these findings raise the possibility that the quick dissociation of the p38 /NPM/PP2a complex in the cytoplasm allows an initial rapid and acute adaptive response of normal endothelial cells to stress-induced injury through F-actin remodeling. For high concentrations of oxidative stress, this initial adaptive response will be associated with a later deregulated response to DNA damage, leading on the long-term to unprocessed DNA damage (Figure 5). This suggests that p38 and its partners could act as a molecular switch that may push cells toward death or survival.

#### p38 and endothelial apoptosis

p38 may trigger either cell death or cell survival programs, depending on cell type, intensity and duration of the activating signal and its crosstalk with other signaling pathways [143]. Notably, p38 has anti-apoptotic effects in tumor cells treated by a genotoxic stress, sustaining the MK2-dependent cytoplasmic sequestration of Cdc25B/C to block mitotic entry and to enhance survival [144]. In endothelial cells, activation of p38 is related to apoptosis after exposure to a large number of stimuli from different origins: physical stress (heat, shear stress, radiation), ligands of death receptors (TNFα, FAS, CD40), pharmacological agents (chemotherapeutics, anisomycin), exogenous H_2_O_2_ [13], 56. The majority of these pro-apoptotic stimuli generate ROS that act as second messengers inducing p38 activation. For example, docosahexaenoic acid (DHA) and arachidonic acid induce apoptosis in HUVEC, which is associated with a decrease of the mitochondrial membrane potential, an increase in ROS generation and the activation of p38 [145]. Accordingly, anti-oxidants as vitamin D [146] or polyphenols like ECGC [147], as well as statins [148] limits ROS generation, p38 activation and endothelial apoptosis. To the same extent, the pharmacological inhibition of NADPH in endothelial cells blocks ox-LDL-induced up-regulation of NADPH oxidase, increase of intracellular ROS, activation of p38 and apoptosis [149]. In response to pro-apoptotic signals, the bioactive sphingolipid ceramide is considered as a major transducer of apoptosis [150]. In the specific context of oxidative stress mediated by high dose of ionizing radiation, ceramide specifically generated through sphingomyelin hydrolysis by the enzyme acid sphingomyelinase (ASMase) has been convincingly described as essential in mediating apoptosis [35, 151]. Endothelial cells from mice defective for ASMase are protected from radiation-induced cell death [152, 153]. The connection between ceramide and p38 phosphorylation during apoptosis has already been shown in several cell models, including cortical neurons treated with C2-ceramide [154], UV-B-irradiated HaCat cells [155] or Fas-activated lymphocytes [156]. Endothelial cells display a 20-fold higher ASMase activity compared to other cells [157] and rapidly generate ceramide in response to ionizing radiation [151]. The molecular mechanisms of ASMase activation are partially elucidated and some studies suggested a redox mechanism, as a direct activation of ASMase by ROS and its inhibition by ROS scavengers have been reported [158, 159]. As mentioned above, p38 is linked to the endothelial apoptotic response. Nevertheless and until recently, the involved molecular mechanisms still needed to be clarified. Our very recent data highlight a new molecular pathway of p38-mediated apoptosis in endothelial cells exposed to ROS-generating stimuli such as ionizing radiation and anisomycin. Activation of ASMase and generation of ceramide initiate this signaling. Then, ceramide, by its specific biophysical properties, coalesces to form ceramide-enriched lipid rafts that drive activation of ASK-1/p38 MAPK, leading to apoptosis [35]. Conversely, ceramide generated by the neutral sphingomyelinase in endothelial cells is not involved in the activation of p38, after cigarette smoke-induced ROS [141]. Nevertheless, in response to endothelin-1 or to oxidized LDL, p38 MAPK activates neutral sphingomyelinase [160, 161].

Endothelial cell apoptosis that depends on p38 is also mediated by transcriptional and post-transcriptional mechanisms. First, mechanisms may imply transcription factors, such as NFκB or p53. Notably, DHA-induced HUVEC apoptosis is under the control of phosphorylation of p38 and p53 at serine 15. To the same extent, ox-LDL induces endothelial cell apoptosis trough ROS-p38MAPK-NFκB signaling cascade and the p53-Bcl-2/Bax-caspase-3 signaling pathway [162]. Second, p38 activation may also modulate the phosphorylation of proteins with pro-survival or pro-apoptotic properties. For example, the activation of p38 induced by TNF-α triggers apoptosis in endothelial cells via phosphorylation and down-regulation of pro-apoptotic Bcl-xL [163]. On the other hand, the expression of anti-apoptotic Bcl2 is associated with inhibition of p38 in response to γ radiation, which may contribute to cytoprotection of endothelial cells, independently of cytochrome c release [164]. Intriguingly, FLIP expression increases the phosphorylation of p38, which in turn increases the phosphorylation of pro-apoptotic Bax leading to inhibition of apoptosis under hyperoxic stress in endothelial cells [165]. This indicates that the apoptotic modulatory functions of p38 may depend on the environmental conditions surrounding endothelial cells.

#### p38 regulates senescence in response to oxidative stress in endothelial cells

Cardiovascular diseases are associated with oxidative stress and accumulation of senescent endothelial cells [166]. Senescence is one of the long-term cellular dysfunctions that contribute to aged-related diseases [167]. Senescent cells are characterized by an irreversible growth arrest that is under the control of the p53 and/or p16^INK4a^ anti-oncogene activation and that leads to respective p21^WAF1^ over-expression and retinoblastoma hypo-phosphorylation. This aging process can be triggered by different mechanisms including oxidative stress, telomere shortening, DNA damage, oncogene signaling, microRNA and mitochondrial dysfunctions [168]. Senescent endothelial cells are dysfunctional, harboring an uncoupling of endothelial nitric oxide synthase (eNOS) and exhibiting increased inflammatory responses with enhanced expression of adhesion molecules VCAM-1 and ICAM-1 [166]. They also acquire an inflammatory secretory phenotype called senescence-associated secretory phenotype (SASP), composed of pro-inflammatory cytokines/chemokines (mainly IL-6 and IL-8), proteases MMPs and several growth factors [169, 170]. In normal senescent fibroblasts, the SASP appears to be largely initiated by NFκB and p38 signaling [171], both at the level of transcriptional and post-transcriptional level [172]. Interestingly, senescence of endothelial cells may also be under the control of p38 signaling. Notably, oxidative stress-induced senescence in HUVECs relies on p38 and is prevented by IL-8 through inhibition of the activation of p38 and NFκB pathways and activation of telomerase activity [173]. In aging senescence endothelial cells, overexpressed Arg-II (L-arginine ureahydrolase arginase-II) induces senescence associated to eNOS-uncoupling and cytokine secretion. Both events depend on p38 activation without a causal link between IL-8 and eNOS uncoupling [174]. Additionally, ionizing radiation induces senescence though the MKK6/p38/SIRT1 and this effect is prevented by pretreatment of proliferating endothelial cells with the antioxidant vitamin D [146]. The Notch signaling pathway also impairs the process of endothelial senescence linked to vascular diseases by inhibiting p16 expression via overexpression of inhibitor of DNA binding 1 (Id1). Conversely, Notch-1 positively regulates the expression of Id1 through MKP-1 phosphatase-induced inactivation of p38 [175]. Additionally, few studies demonstrate that senescence of endothelial cell progenitors (ECP) are triggered by p38 activation in response to TNFα [176]. In those cells, cleaved high-molecular-weight kininogen (HKa), an activation product of the plasma kallikrein-kinin system, induces a concentration-dependent increase in ROS generation associated with up-regulation of p38 phosphorylation and of pro-senescence molecule p16^INK4a^. Conversely, inhibition of p38 prevents HKa-induced EPC senescence [177]. Along the same line, doxorubicin induces p38-dependent EPC senescence that is antagonized by JNK [178]. On the other hand, adiponectin prevents EPC senescence by inhibiting ROS/p38 MAPK/ p16^INK4a^ cascade [179]. Intriguingly, doxorubicin-induced endothelial senescence is not accompanied with a typical senescence secretory response. This is due to the fact that endothelial cells repress senescence-associated inflammation via down-regulation of the PI3K/AKT/mTOR pathway and that reactivation of this signaling axis restores senescence-associated inflammation. Thus, damage-associated paracrine secretory responses are restrained to maintain tissue homeostasis and prevent chronic inflammation [180]. All together, these findings support the view that activation of the p38 cascade is a major pathway that regulates senescence of endothelial cells and thereby that it may contribute to senescent-dependent endothelial and vascular dysfunctions, notably in response to ROS.

In summary, several studies pin point to the fact that the p38 cascade is a major pathway involved in transducing the oxidative stress signal in endothelial cells. In corollary, a better understanding of the endothelial p38 response to ROS is mandatory to impair or delay endothelial dysfunction as well as to protect vessel from age-related diseases.

## THE ENDOTHELIAL P38 PATHWAY AS A MAJOR REGULATOR OF TUMOR PROGRESSION

### Endothelial p38 and tumor progression

Tumors are a mixture of differentially evolved subpopulations of cells in constant Darwinian evolution. These cell subpopulations include endothelial cells, cancer–associated fibro blasts, tumor-associated macrophages (TAM), and immune cells including dendritic cells, T and B cells. With the extracellular matrix (ECM), they constitute the tumor microenvironment that plays a key role during cancer progression, metastasis and therapeutic response [181]. Notably, endothelial cells have essential functions in cancer progression and metastasis by being involved in major essential cancer processes such as hypoxia, angiogenesis and inflammation. Moreover, they are also central components of atherosclerosis-associated cancer. We will briefly review the role of endothelial cell p38 in cancer progression and metastasis.

### Role of endothelial p38 in hypoxia and angiogenesis

Cancer cells cannot survive at a distance greater than 100μm from a blood vessel because they need blood supply to bring oxygen and nutriments required for their survival. In order to grow, cancer cells, within the tumor, initiates the formation of new vessels, mostly following switching-on neovascularization through a process called angiogenesis [182]. Angiogenesis is also centrally involved in metastasis as cancer cells can evade from primary neoplasms and enter into newly formed vessels to colonize distant organs and form metastases. Hypoxia, is one of the major factors that switch-on angiogenesis. Numerous studies have shown that activation of p38 is importantly involved in regulating both hypoxia and angiogenesis.

#### Role of p38 in hypoxia

Solid tumors contain regions at very low oxygen concentrations, a condition called hypoxia. Hypoxia is a feature of most solid tumors in which it contributes to cancer progression by modulating among others angiogenesis and metastasis [183, 184]. The classic process can be summarized this way: cancer cells and their microenvironment resides in hypoxic regions, which triggers the activation of the transcription factor HIF-1 (hypoxia-inducible factor-1α/β) in the cancer cells and also in endothelial cells. In turn, activated HIF-1 initiates the transcription of angiogenic factors including VEGF. As described below, this latter, whether it is produced by paracrine (cancer cells) or autocrine (endothelial cells) ways binds to its receptor VEGFR2 on blood vessel endothelial cells to initiate the signaling cascade leading to angiogenesis. Several studies point to the fact that p38 is importantly involved in hypoxia-induced activation of HIF-1 in cancer cells. For example, it has been shown using cells from p38^-/-^ and MKK3^-/-^ MKK6^-/-^ knockout mice that the p38 pathway is necessary for the hypoxic activation of HIF-1. Moreover, activation of this cascade is initiated from the ROS-generated by the mitochondria complex III during hypoxia [185]. Analogously, chromium (VI), a potent carcinogen, induces the expression of HIF-1 and VEGF in an H_2_O_2_- and p38-dependent manner in DU145 human prostate carcinoma cells [186]. Hypoxia also induces the activation of p38 in squamous head and neck carcinoma cell lines, which results in activation of HIF-1 and VEGF expression [187]. Based on these findings, it appears that p38 signaling is essential for HIF-1 activation and VEGF production by cancer cells and endothelial cells under hypoxic conditions. On the other hand, hypoxia also leads to p38-dependent actin reorganization in endothelial cells, which may be associated with a pro-inflammatory phenotype (increased adhesion of neutrophils to endothelial cells), another hallmark of cancer [188, 189].

#### Role of p38 in angiogenesis

**Figure 6:**
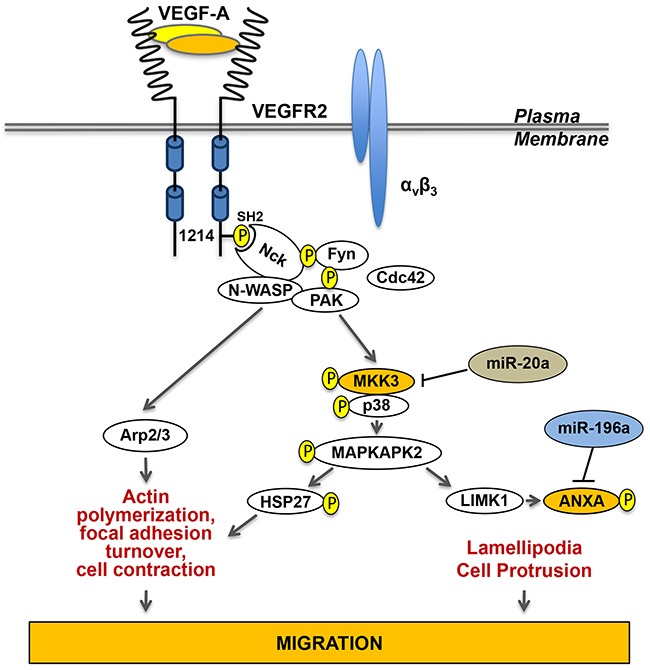
p38-mediated endothelial cell migration in response to VEGF The actin remodeling following the binding of VEGF to VEGFR-2 requires a cooperative interaction between VEGFR-2 and integrins, especially integrin α_v_β_3_. The activation of VEGFR-2 initiates its autophosphorylation at Tyr1214. Then, follows the recruitment of Nck to VEGFR-2 and the sequential activation of Fyn, Cdc42 and PAK upstream of the p38 MAP kinase module. Activation of p38 leads to activation of MAPKAP kinase-2 (MK2), which contributes to increase the level of polymerized actin by phosphorylating HSP27. Interestingly, activation of MK2 also activates LIMK1, which phosphorylates annexin A1 (ANXA) to promote formation of lamellipodia and cell protrusion. Of note, Nck can also trigger actin polymerization through activation of the WASP-Arp2/3 pathway. Both together, actin polymerization associated with stress fibers-mediated cell contraction and lamellipodia-mediated increased cell protrusion contribute to sustained cell migration, an essential step of angiogenesis. (Adapted from 99).

Vascularization occurs via two major mechanisms: vasculogenesis and angiogenesis. Endothelial cells are at the heart of both processes. Vasculogenesis refers to the formation of blood vessels by a *de novo* production of endothelial cells from hemangioblasts, when they are no pre-existing vessels. In contrast, angiogenesis refers to the formation of new blood vessels from pre-existing ones. The latter process is regulated by a tight balance between pro- and anti-angiogenic agents [28]. p38 regulates different steps of the angiogenesis process in different models including *in vivo* mouse models, notably via p38-regulated/activated protein kinase (PRAK) phosphorylation [190]. Notably, besides its involvement in HIF-1 activation, p38 also regulates angiogenesis via its role in endothelial cell migration, an essential step of angiogenesis. The sequence of events is as follows: Hypoxia-induced activation of HIF-1 in cancer cells leads to the expression of VEGFA_165_, one of the 5 spliced isoforms of VEGFA. VEGFA_165_ (herein called VEGF) contains 165 amino acids and it is the most abundant form of VEGF and the most potent pro-angiogenic agent. VEGF binds to its tyrosine kinase receptor VEGFR2 present at the surface of blood vessel endothelial cells. The binding of VEGF to VEGFR2 triggers its oligomerization, which activates its kinase domain and auto-phosphorylation at several tyrosine residues, notably Y1214 [18] Figure 6. Then follows the binding of Nck to p˜Y1214 and the recruitment of Fyn to Nck bound to p˜Y1214 within VEGFR2. Fyn will then initiate a cascade of phosphorylation events involving Nck, and PAK2 located downstream of Cdc42. This will lead to activation of the p38/MK2/HSP27 axis and the subsequent actin remodeling and cell migration [99, 191]. A second mechanism of recruitment of Nck to VEGFR2 involves the nucleation factor N-WASP, which is relocalized at the cell surface by Nck bound to PAK. N-WASP activates the ARP2/3 complex, a major regulator of actin nucleation and stress fibers in motile cells [192]. Moreover, Nck recruitment to VEGFR2 triggers the assembly of focal adhesion via PAK activation, which also contributes to the bundling of actin filaments into stress fibers [193, 194]. Of note, the activation of VEGFR2 requires its association with integrin α_v_β_3_ [195–197]. In summary, VEGF-induced cell migration results from actin remodeling into stress fibers via activation of the VEGFR2-Nck/p38/MK2/HSP27 axis [28], and via VEGFR-Nck-WASP-mediated actin polymerization and focal adhesion turnover [193, 194]. p38 activation by VEGF also contributes to cell migration by formation of lamellipodia via the p38/LIM kinase/annexin-1 axis [62, 100]. On the other hand, degradation of the ECM by metalloprotease (MMP) is another important event that contributes to angiogenesis. In this context, it is also possible that p38 contributes to angiogenesis by regulating the proteolytic activity of MMP (MMP-9 and MMP2/uPA), as reported recently [198, 199] (Figure 6). Overall, by contributing to angiogenesis, activation of endothelial p38 is a key player of tumor neovascularization and thereby of cancer growth and cancer cell dissemination.

### Role of p38 in cancer and inflammation

Inflammation plays an essential role in tumorigenesis and tumor progression and is now recognized as an enabling hallmark of cancer [189]. Along these lines, inflammation plays a central role in the pathogenesis and progression of almost all solid tumors. Accordingly, the notion of inflammation in cancer has greatly changed the approach to treat cancer [200]. On the other hand, the first identified role of p38 was in inflammation [13]. Notably, p38 regulates the production of inflammatory mediators (IL-8, IL-1, IL-6, TNFα, COX-2) [201]. Hence, p38 plays major roles in most cases of cancer-associated inflammation, both at the level of the tumor cell and of the tumor endothelial microenvironment

#### Role of p38 in cancers associated with inflammation

Many cancers arise at sites of chronic irritation and infection associated with inflammation. One of the better known is gastric cancer that frequently originates from *Helicobacter pylori*-induced chronic gastritis and stomach ulcers [202]. Additionally, there is strong evidence that colon cancer can arise from Inflammatory Bowel Diseases (IBD) that include Crohn's disease and ulcerative colitis [203].

Inflammation-associated cancers are characterized by the recruitment of macrophages and neutrophils to cancer sites [204]. These inflammatory cells, particularly the macrophages present within the tumor microenvironment promote ECM degradation and tumor cell motility, which activates endothelial cells and thus enable angiogenesis, Furthermore, a subset of monocytes expressing Tie2, an angiopoietin receptor, are recruited to some cancer sites where they initiate neo-vascularization via different signals including hypoxia [204–206]. Another way by which inflammation contributes to cancer initiation and progression is through the modulation of expression of endothelial adhesion molecules such as a E-selectin, which will catch circulating cancer cells allowing them to roll on the endothelium. This E-selectin-dependent adhesion and rolling is followed by firmer adhesion via molecules such as VCAM and integrins enabling trans-endothelial migration and extravasation [207]. This process occurs in part via activation of endothelial p38-mediated actin contractility and opening of IEJ, as discussed below [208, 209]. Overall, these findings are strong indications that inflammation may act as cancer facilitator and that endothelial cells and p38 are key actors of cancers associated with inflammation.

#### Role of p38 in cancers generating inflammation

Whereas inflammation can contribute to cancer initiation and progression, several findings indicate that cancer cells can themselves generate an inflammatory response that plays a critical role in the cancer microenvironment. Cancer initiates these responses through several mechanisms including transcriptional regulation of inflammatory genes [210, 211]. Notably, several mouse models support the point that p38 is a major player involved in regulating inflammatory cytokines produced by cancer cells [212]. In this context, p38 is required to induce the production of IL-1β and TNFα, two important mediators of inflammation [30, 213] that react with endothelial cells to induce the expression of adhesion molecules. In addition, MK2, the downstream effector of p38, regulates LPS-mediated production of TNFα [213]. Moreover, hypoxia activates tumor-promoting inflammatory response, via recruitment of inflammatory cells such as TAM, T-Regs and neutrophils and thereby plays a major role in mediating cancer-derived inflammation [200].

Overall, these findings are consistent with the view that inflammation is part of an insidious retroactive synergic loop in cancer. Along these lines, the inflammatory responses are upstream of several cancer associated-events such as the recruitment of immune cells, cancer cell proliferation, survival, and angiogenesis [214]. In corollary, there is a toxic synergic overlapping between the pathways involved in both inflammation-associated cancer and cancer-derived inflammatory responses. This is the case, for example, for the role of tumor inflammation in the extravasation process during metastasis. As reported above and described later, inflammation generated from cancer cells can induce the expression of endothelial adhesion molecules such as E-selectin, VCAM and integrins, which will contribute to open IEJ via pathways involving ERK, and p38 and favor extravasation of cancer cells [208, 215].

#### Role of p38 in atherosclerosis–mediated cancer progression

Atherosclerosis, a typical manifestation of endo-thelial dysfunction, shares several common features with cancer. Both pathologies can be induced by similar agents such as oxidative stress, cigarette smoke and increased intake of dietary fats [216]. Moreover, they might have similar causal processes, as atherosclerosis, in a way analogous to what occurs in carcinogenesis, may arise from mutations that transform a single quiescent arterial smooth muscle cell into a proliferative progenitor clone. Additionally, alterations in endothelial cell adhesion molecules such as VCAM are linked both to atherosclerotic plaque formation and to tumor invasion and metastasis. Similarly, altered expression of proteases is associated with plaque expansion and with cancer invasion and metastasis. Cell proliferation pathways involving genes regulating the G_1_/S checkpoint (p53, pRb) are associated with plaque progression after angioplasty as well as with cancer progression. Nuclear transcription factors such as inactivation of NFκB are associated with progression of both diseases [217]. Regulators of angiogenesis including VEGF are not only linked to plaque expansion and restenosis of atherosclerotic lesions but also to local and metastatic tumor expansion. On the other hand, it should be noted that patients suffering from atherosclerosis may respond differently to cancer chemotherapy given the fact the functionality of endothelial cells is altered. Indeed, vascular aging and diseases may affect tumor progression, angiogenesis, and responses to therapy. Accordingly, exposure of tumor-bearing mice to metronomic therapy with cyclophosphamide exerts anticancer effects in young mice, but this effect is reduced in old and atherosclerotic mice, in part due altered angiogenesis [218]. Interestingly, p38 is centrally involved as a pathway that transduces biological responses shared by both atherosclerosis and cancer. These responses include regulation of G1/S checkpoint, activation of NFκB, regulation of cell adhesion molecule, sensitivity to VEGF and ROS. In accordance, p38 may be viewed as a common denominator of both atherosclerosis and cancer progression.

### p38-mediated metastasis

#### The metastatic process

In most cases, death associated with cancer results from the formation of distal neoplasms called metastases. The metastatic process consists of sequential inter-related steps during which cancer cells interact with their microenvironment, namely endothelial cells, cancer associated fibroblasts, macrophages, immune cells and ECM. All the steps must be successfully completed to give rise to metastases [219, 220]. These sequential events include the release of cancer cells from the primary tumor, their intravasation into the blood or lymphatic vessels, their survival in the circulation and their arrest in the capillary bed of a distal organ. In certain circumstances, cancer cells will grow locally within the blood vessels but most of the times they will cross the endothelial layer and extravasate into the surrounding tissue. Then, they will proliferate and initiate neo-vascularisation of the secondary neoplasm. This series of events is destructive to most cancer cells and the formation of metastases is an intrinsically inefficient process [221, 222]. Nevertheless, a small number of them traverse all these steps and form metastases. More specifically, high resolution *in vivo* video-microscopy and cell-fate analysis suggest that the early steps of the haematogenous metastatic process (intravasation into the bloodstream, arrest in the secondary organ and extravasation) are completed very efficiently. In contrast, later steps (growth of micrometastases into the secondary organ, vascularization and persistence to form macroscopic metastases) are rather inefficient [223–226]. This inefficiency may be explained by the fact that micrometastases are established by tumor stem cells, that are few in number, and by the fact that the invading cells quickly fall into apoptosis [221, 227, 228]. Interestingly, several studies have reported the contribution of p38 in transducing signals that participate to the metastatic process either in endothelial cells and cancer cells.

#### p38 signaling in endothelial cells during metastasis

Endothelial cells have key functions during the metastatic process. As mentioned above, cancer cells shed from a primary tumors must cross the endothelium to enter the circulation during the intravasation process. Thereafter, circulating cancer cells must re-cross the endothelium again during the extravasation step to come out the vessels and colonize another sites. In both cases, this involves adhesive interactions between adhesive receptors present on both endothelial cells and on cancer cells.

#### Adhesion of metastatic cancer cells to endothelial cells

Adhesion of cancer cells to endothelial cells involves their binding to endothelial cells via adhesion receptors such as E/P-selectin, VCAM, PECAM and integrins. Notably, E-selectin mediates the adhesion of tumor cells to endothelial and this interaction is associated with metastatic dissemination [207, 229–235]. For example, highly metastatic human colorectal and mouse lung carcinoma cells, upon their entry into the hepatic microcirculation, trigger a rapid host pro-inflammatory response by inducing TNFα production in resident Kupffer cells. In turn, this triggers E-selectin expression by endothelial cells and enhances the binding and extravasation of the cancer cells [215, 230]. Along these lines, the binding efficiency of clonal colon cancer cell lines to E-selectin on endothelial cells is proportional to their metastatic potential [236]. Additionally, the E-selectin expression induced by the pro-inflammatory cytokine IL-1β is directly and negatively regulated by miR-31. The miR-31-mediated repression of E-selectin in endothelial cells impairs the metastatic potential of colon cancer cells by decreasing their adhesion to the endothelium. The transcription of miR-31 is activated by IL-1β and depends on p38 and JNK, and their downstream transcription factors GATA2, c-Fos and c-Jun [85].

**Figure 7:**
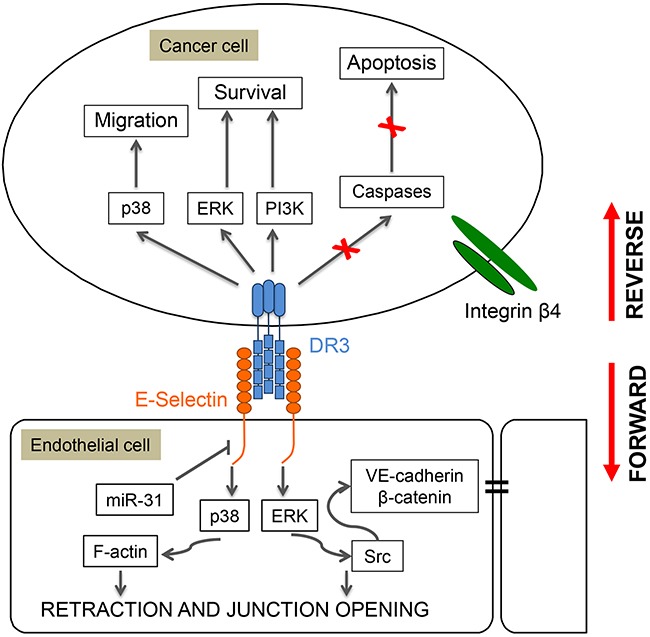
Role of p38 in reverse and forward signaling induced by E-selectin in cancer cells and endothelial cells Adhesion of colon cancer cells to endothelial cells expressing E-selectin induces a reverse signaling in the cancer cells that increases their motile potential, and a forward signaling in the endothelial cells that increases inter-endothelial permeability and enables extravasation. For example, adhesion of colon carcinoma cells to endothelial cells involves the binding of E-selectin on endothelial cells to Death Receptor-3 (DR3) on cancer cells. This interaction induces the reverse activation of p38, ERK and PI3 kinases in cancer cells, which increases their motile and survival potentials. In contrast, caspases (3 and 8) are not activated and caspase-dependent apoptosis is impaired. Reciprocally, the interaction between DR3 and E-selectin triggers the forward activation of the same MAP kinase pathways in endothelial cells. This results in myosin-light chain (MLC)-mediated cell retraction and in dissociation via ERK and Src activation of the VE-cadherin/β–Catenin complex and thereby destruction of *adherens* junctions leading to increased endothelial permeability and extravasation of cancer cells.

The adhesion of several types of cancer cells to endothelial E-selectin requires oligosaccharide/protein complexes known as E-selectin counter-receptors or ligands on tumor cells [209, 235, 237]. In colorectal cancer cells, sialyl Lewis -a and –x are considered to be the representative oligosaccharides involved in E-selectin binding [233, 238–240]. The glycoprotein ESL-1, present on myeloid cells, was the first proteinic component of a counter-receptor to be described for E-selectin [241]. This is a variant of the tyrosine kinase FGF glycoreceptor, raising the possibility that its binding to E-selectin elicits signaling in the adhering cancer cells [241, 242]. Accordingly, the binding of E-selectin to cancer cells triggers the tyrosine phosphorylation of several proteins including Src, c-Cbl, FAK and p38 [243–247]. Among several E-selectin ligands [248–252], Death Receptor-3 (DR3) is a signaling receptor for E-selectin in metastatic colon carcinoma cells [253] that can induce a rapid activation of p38 that increases the motile potential of cancer cells and favors their transendothelial migration [246, 253]. Concomitantly, DR3 binding to E-selectin/Fc chimeras is associated with survival of metastatic colon cancer carcinoma cells via the activation of the ERK and PI3-K pathways [253, 254] (Figure 7). In contrast, caspases (-3 and -8) are not activated and apoptosis is impaired [253, 254]. On the hand, the binding of cancer cells bearing DR3 to E-selectin induces endothelial cell retraction via p38 activation and *adherens* junction opening via disruption of the VE-cadherin-β-catenin complex subsequently to Erk and c-Src activation. Interestingly, the binding of Colo-320 human adenoma to P-selectin triggers the formation of a complex between p38 and PI3K which results in an increased spreading of the cancer cells [255]. This suggests that P-selectin may also be involved in a mechanism that promotes initial attachment of cancer cells to endothelial barrier and favors arrest of cancer cells from the blood flow before extravasation [255].

Overall, endothelial adhesion molecules are major determinants of hematogeneous tumor metastasis, being required for the binding of cancer cells. In turn, this leads to activation of the p38 pathway both in the cancer cells and endothelial cells to trigger and increase the motile and invasive potentials of cancer cells.

#### Transendothelial migration of metastatic cancer cells

The modification of the vascular permeability and consequent disruption of the endothelial barrier contributes to the extravasation of cancer cells and to the promotion of metastasis. In this context, the adhesion of cancer cells to endothelial cells initiates a series of events that enable the cancer cells to breach the endothelial layer and colonize secondary sites. Notably, following their adhesion to endothelial cells, cancer cells extend invadipodia into the endothelial cell junctions initiating their retraction and enabling their transendothelial migration (TEM). The retraction of endothelial cells following E-selectin-mediated adhesion of colon cancer cells expressing DR3 results from an ERK-dependent dissociation of the VE-cadherin/β-catenin complex associated with a p38-dependent retraction of actin filaments [208, 256]. These processes facilitate TEM of colon cancer cells by opening IEJ [208, 256]. More recently by using E-selectin cross-linking and beads coated with CD44 it was shown that CD44/selectin ligation is responsible for ICAM-1 upregulation in HUVECs [257]. The authors present evidence showing that CD44/selectin binding signals to ICAM-1 up-regulation on endothelial cell surface occurs through a PKCα–p38–SP-1 pathway, which further enhances the adhesion of melanoma tumor cells to endothelial cells during metastasis. Very interestingly, Death Receptor 6 (DR6: TNFRSF21)) expressed on endothelial cells has been identified as a key player of TEM and metastasis. Notably, B16 melanoma cells induce the death of endothelial cells by necroptosis, a newly discovered pathway of regulated necrosis, which enables migrating cancer cells to cross the endothelial-cell barrier and colonize the lung [258]. Necroptotic endothelial-cell death is induced by interaction of amyloid precursor protein on the tumor surface with DR6 on endothelial cells. In turn, this interaction enhances TEM 1) directly, as a consequence of endothelial-cell death and disruption of the endothelial barrier, or 2) indirectly, via the release of damage-associated molecules from dying endothelial cells, which could open the endothelial barrier between cells. On the other hand, DR6 is also required for tumor angiogenesis in a B16 xenograft mouse model by preventing angiogenesis [259]. This effect has been attributed to the fact that DR6 is required for the production of IL-6, which in turn will increase the production of angiogenic agents (VEGF, PDGF) in a p38-dependent manner. Hence, DR6 is required for the induction of angiogenesis and eventually of metastasis associated with TEM and extravastion of cancer cells across newly formed blood vessel and it involves the IL-6/p38 MAPK pathway. Along this line, the role of paracrine mediators secreted by tumor cells in regulating melanoma metastasis has been recently reinforced as the binding of the melanoma tumor-secreted protein SPARC to endothelial VCAM drives endothelial permeability and cancer cell extravasation through a ROS-MKK3/6-p38 MAPK pathway [260].

Together, these studies suggest that the binding of cancer cells to endothelial cells induces both a forward signaling in the cancer cells and a reverse signaling in endothelial cells (Figure 7). The forward signaling induces p38-dependant actin remodeling underlying cancer cell adhesion and migration, but also a p38-dependent production of angiogenic paracrine factors. The reverse signaling enables transendothelial migration of cancer cells via dissolution of *adherens* junctions through p38-increased actin contractility and opening of IEJs and but also via physical defect of the endothelial barrier as a consequence of endothelial cell necroptosis.

#### p38 in lymphatic system-associated metastases

Several metastatic cancer cells including breast cancer transit through the lymphatic system during metastatic dissemination. Notably, mammary tumor cells undergoing Epithelial Mesenchymal Transition (EMT) in response to TGF-β1 are targeted for migration preferentially through the lymphatic system [261]. This relies on the capacity of TGF-β1 to promote crosstalk between cancer cells and lymphatic endothelial cells via CCR7/CCL21. This occurs as follows: 1) TGF-β1 promotes CCR7 expression in EMT cells through p38-mediated activation of JunB, 2) TGF-β1 promotes CCL21 expression in lymphatic endothelial cells. In turn, CCL21 favors the chemotactic migration of cells undergoing EMT toward lymphatic endothelial cells [261]. These findings suggest that inhibition of p38 may be a suitable approach to inhibit EMT and lymphogenic dissemination of tumor cells. Moreover, in human primary melanoma cells, a high level of VEGF-C and a low level of MITF (microphtalmia-associated transcription factor) correlate in a p38-dependent manner with an increase risk of metastasis associated with lymphangiogenesis [262]. In fact, p38 MAPK signaling constitutes a major pathway regulating migratory properties of EMT cells since it is centrally involves in cross-talking with other pathways that are known to cooperate to induce EMT including by JunB and TGF-β [261, 263]. Here again, these studies indicate the primary role played by p38 in the metastatic process.

### p38-as a modulator of cancer treatment

As described above, the p38 pathway is a pleiotropic cascade that plays a central role in the functioning and dysfunctioning of endothelial cells in response to growth factors and stress and during carcinogenesis and metastasis. In this context, endothelial p38 has major impact on conventional therapeutic approaches, namely radiotherapy and chemotherapy.

#### Impact of endothelial p38 in radiotherapy

More than 50% of cancer patients are treated with ionizing radiation. The importance of the endothelial compartment in the response of tumor but also of normal tissues to ionizing radiation has been highlighted over the past 10 years [152, 153, 264]. Exposure of the microvasculature to radiation provokes acute and late effects, both of which contribute to the initiation, progression and maintenance of tissue damage [265]. Early effects of radiation lead to a rapid wave of endothelial apoptosis or to a dysfunctional vascular phenotype marked by excessive secretion of pro-inflammatory cytokines, increased recruitment of blood cells and platelets, activation of the coagulation system and increased vascular permeability [265]. Late effects include collapse of microvessels, thickening of the basement membrane and persistence of an activated pro-coagulant endothelial phenotype, that ultimately may become senescent [266]. Ionizing radiation-induced molecular signaling remains insufficiently characterized in endothelial cells. Nevertheless, the involvement of the p38 MAPK pathway has been described in several endothelial cells models. In human dermal microvascular endothelial cell, ionizing radiation-induced apoptosis is predominantly mediated through p38 [267] and can be inhibited by inhibition of the p38 pathway through Bcl2 overexpression [164]. Recently, a new signaling pathway that depends on p38 has been revealed by findings showing the implication of plasma membrane reorganization in radiation-induced apoptosis [35]. Notably, ionizing radiation-induced activation of p38 in microvascular endothelial cells requires the upstream activation of the sphingolipid ASMase/ceramide pathway. Upon exposure to radiation, the pool of ceramide generated leads to plasma membrane reorganization associated with the formation of ceramide-enriched lipid signaling platforms, which, in turn, contributes to p38 activation and induction of apoptosis. Activation of p38 in response to ionizing radiation is also associated with an increased secretion of pro-inflammatory cytokines by endothelial cells. In particular, radiation-induced IL-6 appears to be controlled by p38 through activation of NFκB [268] and radiation-induced endothelial expression of IL-6, IL-8, CXCL-2 and E-selectin, exacerbated by the interaction of endothelial cells with immune mast cells, is also dependent on p38 [269]. As described previously, exposure to ionizing radiation induces not only apoptosis but also senescence, especially in quiescent endothelial cells [270]. Senescent endothelial cells exhibit cell cycle arrest, increased expression of adhesion molecules, elevated production of inflammatory cytokines and ROS, and decreased production of NO [271]. The role of p38 in promoting the cell cycle arrest through the p53/p21/p16 axis as well as in regulating pro-inflammatory cytokines present in the SASP may also be considered as contributing to endothelial senescence. Additionnally, MKK6/p38 has been reported to mediate radiation-induced endothelial senescence through downregulation of the sirtuin SirT1 [146]. Overall, these findings suggest that p38 may be a major determinant of the therapeutic response of cancer patients treated by ionizing radiation.

#### Impact of p38 in chemotherapy

Apoptosis is a major mechanism of cell death in response to anticancer agents and several studies already reported the role of p38 in modulating the chemotherapeutic response of both conventional treatment and novel agents including tyrosine kinase inhibitors (TKI) as well as monoclonal antibodies [272]. p38 has been implied in cell apoptosis mediated by cis-platinium and 5-fluorouracil (5-FU) in breast and colon cancer cell lines [272]. Furthermore, inhibition of p38 has been associated with resistance to gemcitabine and cytarabine [272, 273].

On the other hand, p38 contributes to resistance to chemotherapies in several types of tumors. In response to doxorubicin, cis-platinium or DNA-damage agents, p38 favors cell survival in p53-deficient cancer cells through MK2-mediated cell cycle arrest [144, 274]. Indeed, inhibition of p38 in colon and breast tumor mouse models potentiates the effects of conventional chemotherapies like cis-platinium, 5-FU or irinotecan [275]. Furthermore, mouse hepatocarcinoma are sensitized to treatment by the TKI sorafenib, when p38 is inhibited [276]. Both p38-mediated resistance and sensitivity have been reported following treatment with 5-FU and cis-platinium (see review [272]). The role of p38 in the chemotherapy response appears to depend on the type of tumor, but might also be regulated by the tumoral microenvironment.

The contribution of p38 in tumor progression involves different cell types within the tumor microenvironment, as described in the above sections. Notably, considering the endothelial compartment as an essential component of the cancer microenvironment and its role in angiogenesis, several inhibitors of the p38 pathways show interesting potential in inhibiting cancer progression via inhibition of angiogenesis. For example, pharmacological inhibition of endothelial p38 is associated with a significant reduction in tumor growth and in vessel density in an *in vivo* experimental model of prostate cancer [277]. On the other hand, p38 inhibition in head and neck squamous cell carcinoma reduces cancer growth in tumor xenografts and underlies a marked decrease in intratumoral angiogenesis affecting both blood and lymphatic vessels [278]. Some known anti-angiogenic agents act directly on the endothelial p38 pathway or on p38 expressed in cancer cells by impeding them to induce angiogenesis, and could be considered as anti-angiogenic agents. Inhibition of p38 leading to angiogenesis inhibition emerges as a potential target to impair cancer progression and metastasis [99]. Given the importance of p38 in driving angiogenesis, one may expect the development of agent that will specifically target p38 to inhibit angiogenesis and metastasis.

Endothelial p38 also contributes in different ways to modulate the response of cancer cells to chemotherapy. For example, genotoxic stress induced by ROS-generating drugs such as doxorubicin triggers the acute release of IL-6 from thymic endothelial cells in a p38-dependent manner. In turn, this acute secretory response precedes the gradual induction of senescence in tumor-associated stromal cells [180, 279]. The inflammatory secretome of senescent stromal cells (SASP) is an additional microenvironmental factor, promoting tumor progression that is regulated by p38 [172]. In this context, inhibition of p38 has been shown to compromise the pro-tumorigenic capacities of the microenvironment.

Overall, the importance of targeting p38 cancer cells and endothelial cells in cancer chemotherapy is supported by several studies. These studies open new perspectives in cancer treatment, as it will be discussed in the next section.

## CONCLUDING REMARKS

Initially described as a major signaling cascade that transduces stress responses, the p38 pathway appears as a pleiotropic pathway regulating the cardiovascular system [280]. p38 is a major contributor to the regulation of endothelial cell response to ROS generated either by inflammation, aging, cancer-derived paracrine factors, radiotherapy or by chemotherapy. This places p38 as a cornerstone of the oxidative stress response in endothelial cells. Accordingly, deregulated p38 signaling in response to ROS is associated with dysregulation of endothelial permeability and alteration in DNA Damage Response, which leads to endothelial dysfunction and ultimately to various associated pathologies including atherosclerosis and diabetes. On the other hand, p38 is also importantly involved in ROS-induced apoptosis and senescence. These findings suggest that it is mandatory to understand the mechanisms underlying the p38 response of endothelial cells to ROS in order to delay endothelial dysfunction as well as to protect vessel from age-associated diseases.

p38 activation is also involved in tumor development via endothelial engagement into angiogenesis, either via a primary role in the induction of the tumor angiogenic switch or in angiogenesis-dependent metastatic events. p38 signaling is further deeply involved in a reverse and forward signaling between cancer cells and endothelial cells, through adhesion receptor E-selectin/E-selectin ligand interaction during metastasis. Notably, its activation in cancer cells increases their motile potential whereas its reverse signaling in endothelial cells dissociates the *adherens* junctions enabling transendothelial migration and extravasation of the motile cancer cells, both events enhancing metastasis. p38 signaling also remodels the stromal microenvironment of cancer cells towards a pro-tumorigenic soil, through transcriptional and post-transcriptional regulation of the stromal senescence-associated secretory phenotype (SASP) [172].

Interestingly, p38 by its pleiotropic functions in both tumor cells and cells in the tumor microenvironment appears as a potential important target in cancer therapy. Accordingly, there are about 60 clinical trials evaluating the role of p38 as a biomarker and as a target in different diseases. Fourteen of them are devoted to cancer treatment and in half of them, p38 is viewed a promising target either by using specific p38 inhibitor alone or in combination with other chemotherapeutic agents [272]. In particular, the SOLSTICE trial, a phase 2 randomized trial performed on 535 patients was designed to investigate, the clinical efficacy of losmapimod in myocardial infarction. The results do not show clear major convincing effects of losmapimod-mediated inhibition of p38 on myocardial infarction [281]. Nevertheless, they indicate that inhibition of p38 with oral losmapidod was well tolerated in patients with non-ST-segment elevation myocardial infarction and might improve the outcomes after acute coronary syndromes. These findings were considered as sufficiently clear to move to a much larger phase 3 trial (Latitude-TIMI-60) with clinical end points, such as evaluation of type-1 myocardial infarction [282]. Overall, this trial indicates that the wheel is now running and it leads to high expectations regarding the possibility to inhibit p38 in treating cardiac and eventually other diseases.

Based on all these findings, one may envision that targeting the p38 pathway may reveal to have clinical impact in a near future in the treatment of different pathologies especially inflammatory diseases and cancer. For the moment, several questions remain to be answered, namely in relation with the fact that p38 regulates a large number of biological activities so that its inhibition may be associated with a large spectrum of secondary and major toxic effects. So, a major concern is to develop agents that may specifically target p38 or a specific event that is dysregulated downstream of the pathway and that is responsible for a given disease. In this context, one may envision to develop small peptides aimed at specifically inhibit the interaction of Y1214 within VEGFR2 with its direct adapter protein, thus inhibiting specifically the p38 activation by VEGF, whilst keeping its activation possible by other stimuli. In such a case, it should be possible to specifically inhibit p38-dependent angiogenic events. Another avenue is to develop agents that inhibit the endothelial p38 signal via disruption of p38 from interacting partners such as nucleophosmin. The combination of p38 inhibitors with conventional chemotherapeutic drugs is also a promising new avenue to treat some tumors. In this context, an oral p38 inhibitor, LY2228820/Ralimetinib, characterized as an anti-angiogenic *in vitro* and *in vivo* [283], has been tested in a phase I study in combination with tamoxifen in advanced cancer patients [284]. In this trial, Ralimetinib, showed acceptable safety, tolerability and pharmacokinetics for patients with advanced breast cancers. Additionally, 21.3% of the patients achieved stable disease without progression. Interestingly, Ralimetinib in combination with gemcitabine and carboplatin is under investigation in a double blind, randomized phase II trial for women with platinum-sensitive ovarian cancer (https://clinicaltrials.gov/ct2/show/NCT01663857). Resistance to treatment has to be taken into account when considering cancer therapy and limiting resistance is a major challenge. In this context, inhibition of the p38 pathway limits resistance to irinotecan in colon adenocarcinoma [285]. However, it is difficult to predict which type of tumors would benefit from these combined therapies. Predictive biomarkers would be very useful to select the patients who might benefit from the p38 inhibitors.

In conclusion, important progresses have been done during the last 25 years in the understanding of the regulation and functions of p38. These progresses have been reached through multidisciplinary and translational approaches and we have no doubt that the next coming years will reveal to be fruitful in developing new drugs that will target p38 to improve therapeutic efficacy in diseases such cancer and cardiovascular pathologies.
